# Dislodgement of port-A catheters in pediatric oncology patients: 11 years of experience

**DOI:** 10.1186/1477-7819-11-191

**Published:** 2013-08-13

**Authors:** Su-Chen Wang, Chia-Hui Tsai, Chiu-Ping Hou, Shin-Yi Lee, Sheung-Fat Ko, Chih-Chen Hsiao, Yu-Chieh Chen, Jiin-Haur Chuang, Jiunn-Ming Sheen

**Affiliations:** 1 Department of Pediatrics, Chang Gung Memorial Hospital – Kaohsiung Medical Center, Chang Gung University College of Medicine, Niao-Sung, Kaohsiung 833, Taiwan; 2 Department of Pediatric Surgery, Chang Gung Memorial Hospital – Kaohsiung Medical Center, Chang Gung University College of Medicine, Niao-Sung, Kaohsiung 833, Taiwan; 3 Department of Radiology, Chang Gung Memorial Hospital - Kaohsiung Medical Center, Chang Gung University College of Medicine, Niao-Sung, Kaohsiung 833, Taiwan

**Keywords:** Children, Dislodgement, Port-A catheter, Transcatheter retrieval

## Abstract

**Background:**

Port-A catheters are frequently used in pediatric cancer patients. Their dislodgement is potentially seriously risky although the incidence is not high. We analyzed our 11 years of data to address this important problem.

**Methods:**

From January 2001 to December 2011, 330 port-A catheters of different brands were implanted in pediatric cancer patients. In total, eight children suffered a dislodgement of their catheter. Their ages ranged from four to thirteen years, with a median age of ten. Five patients presented with catheter dysfunction, two presented with a cough and one was identified incidentally during surgery to remove his port.

**Results:**

The downstream ends of the dislodged catheters were located in the right atrium (three patients), left pulmonary artery (three) and inferior vena cava (two). Six of the eight catheters were broken at the site of anastomosis to the port and the other two were broken halfway in between. All episodes of dislodgement happened after the chemotherapy regimen was completed. The dislodged catheters were successfully retrieved without complications by transcatheter retrieval using a gooseneck snare.

**Conclusions:**

The dislodgment rate of port-A catheter in our series was 2.4%. Chest X-rays can rapidly detect the problem. Most of the catheters were broken at the site of anastomosis. Earlier explantation of port-A catheters after completing chemotherapy may be considered to avoid the dislodgement of catheters, but this needs to be weighed against the possibility of underlying disease recurrence. However, we should re-examine how long port-A catheters need to be retained after chemotherapy considering the improved cure rate of pediatric cancer.

## Background

Totally implanted central venous access catheters (for example, the Port-A-Cath) can provide reliable, long-term vascular access for chemotherapy, total parenteral nutrition and frequent blood sampling [1]. They are commonly used in patients with hematologic or oncologic disorders. They significantly improve the quality of life for pediatric oncologic patients with difficult intravenous access [1].

Complications, such as infection and thrombosis, with port-A catheters are not uncommon [2]. The dislodgement and migration of a catheter is a rare but potentially serious complication. In this study, we investigated the incidence and clinical presentation as well as the risk factors of catheter dislodgements to prevent their occurrence.

## Methods

We reviewed the medical records of patients who received replacement port-A catheters from 1 January 2001 to 31 December 2011, and followed up the clinical course until 30 June 2012. This study was approved by the Institution Review Board of the hospital. There were 330 totally implanted catheters of different brands implanted in 297 pediatric cancer patients. Of these, twenty-six patients had a catheter implanted twice, two patients three times and one patient four times.

In total, eight children suffered dislodgement and migration of a catheter. Five patients presented with catheter dysfunction, two presented with a cough and the other one was identified incidentally during surgery for removal of his port-A catheter. All dislodged catheters were successfully retrieved without complication by transcatheter retrieval through the femoral route using a gooseneck snare and one with additional pigtail help. We used chi-squared or Fisher’s exact tests to compare categorical variables. We used Student’s *t* test to compare continuous variables. A *P* value less than 0.05 was considered statistically significant.

## Results

**Figure 1:**
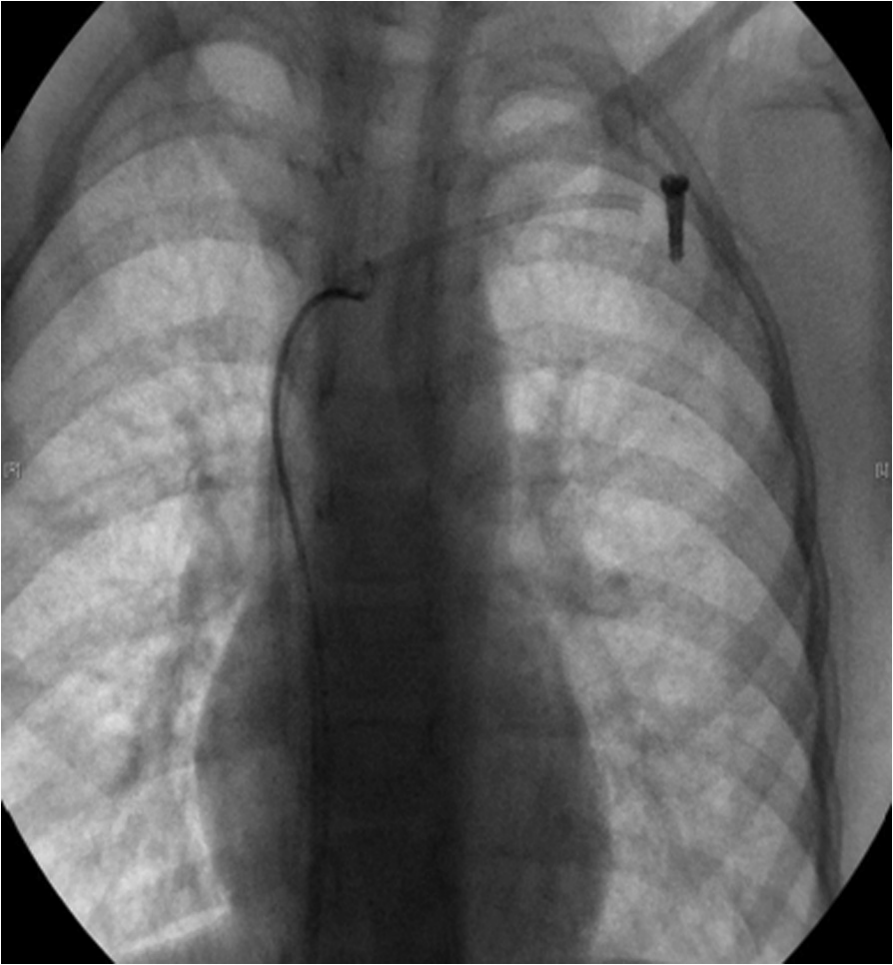
**Chest radiogram in case 7 showing the port-A catheter broken at the site of anastomosis to the port.** The loop of the gooseneck snare has caught the dislodged catheter.

**Figure 2:**
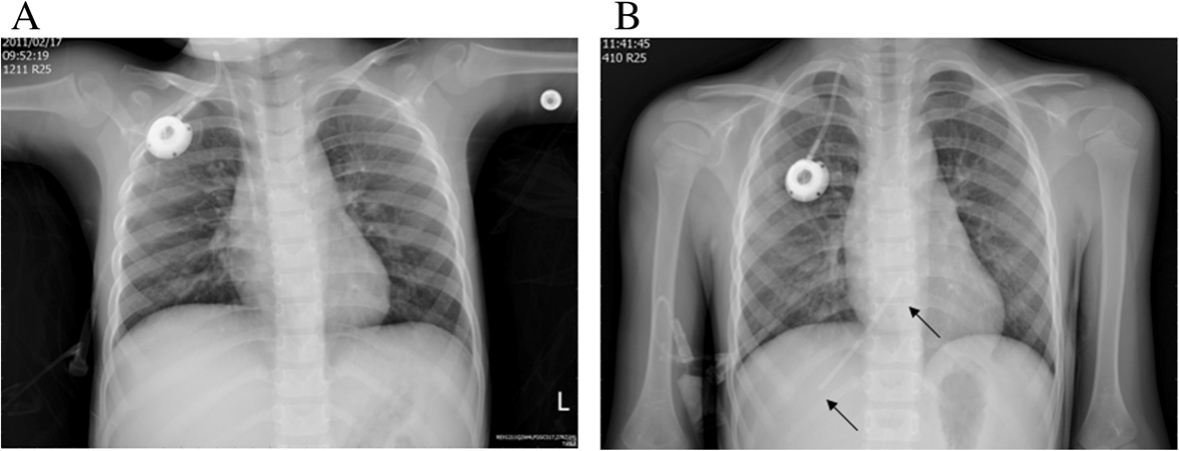
**Chest radiograms for case 8. (A)** Port-A-Cath catheter before dislodgement. **(B)** The catheter broke halfway to the port and the fragment migrated to the hepatic vein and right atrium (arrows).

The data for the patients suffering catheter dislodgements and migration are summarized in Table 1. There were five girls and three boys. Their ages ranged from 4 to 13 years, with a median of 10 years of age. Their body weights ranged from 17 to 78 kg, with a median of 27 kg. Four patients were diagnosed with acute leukemia, two with sarcoma, one with lymphoma and one with immature teratoma. The duration of catheter use was from 5 to 47 months, with a median of 34 months. All episodes of dislodgement happened after the chemotherapy regimen was completed. These patients had all been off chemotherapy for a median of 9.5 months (range 1 to 34 months). Six catheters were located over the right side, two on the left side. The puncture sites were the internal jugular vein in four children, the subclavian vein in three and the external jugular vein in one. There were dislodgements for all four brands and brands Arrow and Bard had three each. The downstream ends of the dislodged catheters were located in the right atrium (three patients), left pulmonary artery (three) and inferior vena cava (two). Six of the eight catheters were broken at the site of anastomosis to the port and the others were broken halfway to the port (Figure 1 and Figure 2).

**Table 1:** Clinical data for eight patients with catheter dislodgment

**Case**	**Age (years old)**	**Sex**	**Body weight (kg)**	**Underlying disease**	**Duration used/off chemotherapy (months)**	**Laterality**	**Vein (internal jugular = 1, external jugular = 2 subclavian = 3)**	**Brand (Bard = 1, Arrow = 2, Brovic = 3, Vortex = 4)**	**Symptoms/signs**	**Location (upstream – downstream)**	**Site of dislodgement**
1	4	F	19	Lymphoma	5/1	R	2	1	Cough	RPA – LPA	Middle
2	12	F	34	ALL	37/8	R	3	2	Dysfunction	SVC – IVC	Anastomosis
3	6	M	25	ALL	47/16	R	1	1	Incidentally at operation	SVC – RA	Anastomosis
4	13	M	56	AML	45/34	L	3	2	Cough, fever, incidentally	RV – LPA	Anastomosis
5	13	M	78	AML	38/9	R	1	1	Dysfunction	LPA – LPA	Anastomosis
6	9	F	17	US	31/18	R	1	3	Dysfunction	Right brachiocephalic vein – IVC	Anastomosis
7	9	F	28	RMS	26/10	L	3	2	Dysfunction	Left brachiocephalic vein – RA	Anastomosis
8	5	F	18	IT	17/4	R	1	4	Dysfunction	Hepatic vein – RA	Middle

*ALL*, acute lymphoblastic leukemia; *AML*, acute myeloblastic leukemia; *F*, female; *IT*, immature teratoma; *IVC*, inferior vena cava; *L*, left; *LPA*, left pulmonary artery; *M*, male; *R*, right; *RA*, right atrium; *RMS*, rhabdomyosarcoma; *RPA*, right pulmonary artery; *RV*, right ventricle; *SVC*, superior vena cava; *US*, undifferentiated sarcoma.

Next, we compared the clinical characteristics of the eight patients who suffered a catheter dislodgement and 322 patients who did not (Table 2). There was no significant statistical difference in sex, age, duration of use, diagnosis, laterality, vein inserted and operating surgeon between these two groups. Only the port-A brand (Arrow to Vortex) had a significant difference. Because all episodes of dislodgement in our patients happened after the chemotherapy regimen was completed, we compared the relation between chemotherapy status and port-A-catheter dislodgement. We found the off chemotherapy group had a higher dislodgement rate statistically. For the 190 patients off chemotherapy, there was no difference between the ‘off chemotherapy less than half year group’ and ‘off chemotherapy longer than half year group’ (Table 3).

**Table 2:** Clinical characteristics of the patients with or without catheter dislodgement

	**Without**	**With**	***P *value**
	**dislodgement**	**dislodgement**	
	**(*N*** **= 322)**	**(*N*** **= 8)**	
Age at insertion (months)	90.2 ± 3.5	82.4 ± 12.5	0.565
Sex (number) (M:F)^a^	189 : 133	3 : 5	0.402
Duration of use (months)	33.9 ± 1.2	32.1 ± 5.0	0.818
Diagnosis			0.089
ALL	152	2	
AML	28	2	
Lymphoma	37	1	
Brain tumor	24	0	
Soft tissue sarcoma	15	2	
Other	66	1	
Port-A brand			0.006^b^
Vortex	134	1	
Bard	151	3	
Arrow	16	3	
Other	21	1	
Laterality (R:L)^a^	248:74	6:2	1
Vein inserted			
Internal jugular	241	4	0.286
External jugular	29	1	
Subclavian	48	3	
Other	4	0	
Operator			0.843
A	181	4	
B	122	4	
C	14	0	
D	5	0	

*ALL*, acute lymphoblastic leukemia; *AML*, acute myelogeneous leukemia; *F*, female; *L*, left; *M*, male; *R*, right.

^a^ Sex and laterality are expressed as ratios, other parameters are expressed as means ± standard error of the mean.

^b^ Arrow to Vortex: *P* = 0.006.

**Table 3:** Relation between chemotherapy status and port-A catheter dislodgement

**Factor (number of patients with dislodgement/number of patients without)**	**% vs.%**	***P *value**
Under chemotherapy (0/330) vs. off chemotherapy (8/190)	0 vs. 4.2	< 0.001
Off chemotherapy less than 1/2 year (2/19) vs. > 1/2 year (6/171)	10.5 vs. 3.5	0.184

## Discussion

The rate of dislodgement of port-A catheters is 1.4% to 3.6% in children [3,4], which is higher than the 0.3% to 1.5% in adults [5,6]. In our study, the rate is 2.4%, which is similar to previous research. For a child, the surgeon usually cuts the catheter and connects it to the port because the body size varies in pediatric patients, which might account for the higher incidence of dislodgment in children, especially at the site of anastomosis, a frequent site of dislodgement [7]. Nevertheless, the period of use has to be taken into consideration because children with cancer have a better survival rate and a longer treatment period than adult patients.

In theory, any dislodgement of a catheter should cause catheter dysfunction. The dislodgement for one patient in our series, however, was found incidentally during surgery to remove his port and two patients in our series presented with coughing. This may be because the patients did not receive regular flushing of the port when the catheter was not being used. Although most dislodgements present with only irrigation resistance or even without symptoms or signs [8], critical conditions such as ventricular tachycardia secondary to port-A fracture and embolization still need to be watched for [9]. We have no exact data that would indicate the percentage of port dysfunction caused by catheter dislodgement but chest radiography can validly detect catheter dislodgment. Chest radiography was needed to be taken before surgery for removal of a port to avoid dislodgement just found during operation in those without regular flushing.

The downstream ends of the dislodged catheters were located in the right atrium (three patients), the left pulmonary artery (three) or the inferior vena cava (two). Tsai et al. reported that the most frequent location of dislodged port-A catheters was between the right atrium and inferior vena cava [10]. The most commonly used retrieval set is the loop snare. Cheng et al. reported that the success rate for percutaneous retrieval of the dislodged fragment was 97.8% and the complication rate was only 3.3% [8]. Therefore the retrieval of dislodged port-A catheters using an endovascular approach might be the first choice of treatment because it is both safe and effective.

The causes of catheter dislodgment include poor connection to the port, catheter damage at the site of anastomosis, incorrect catheter position, catheter damage by chemotherapeutic drugs and the pinch-off syndrome [11,12]. The pinch-off syndrome occurs when a subclavian catheter passes between the clavicle and the first rib and becomes compressed or kinked [13]. Over time, the catheter may become damaged or even broken. This syndrome occurs in up to 40% of adult patients with catheter dislodgments [13]. In our series, all six catheters that dislodged at the anastomotic site between the port and the catheter were damaged and torn at the fracture site, probably owing to material fatigue after long-term use, angulation or exposure to chemotherapeutic drugs. A short residual piece of catheter connected to the port was noted during surgical removal. Furthermore, our cases ranged between 4 and 13 years old and were growing continuously. A tensile force may further draw the catheter out from the port. Therefore, a catheter should be slack between the port and its flexed portion in order to cope with the child’s physical growth. Only two of the broken catheters were not broken at the anastomosis site. Neither of these two broken catheters was punctured at the subclavian vein, indicating classical pinch-off syndrome is not likely the cause of catheter broken. We also found that these two catheters had broken earlier than the other six catheters. Whether the catheters were broken by kinking or compression while turning the head is to be determined.

Chang et al. stated that a cephalic vein cut-down performed by a general surgeon had a lower risk of catheter fragmentation than a subclavian vein puncture through the Seldinger technique performed by a vascular surgeon [12]. Wu et al. also indicated that intravenous port implantation via the subclavian route and the Arrow Fr. 8.1 port were found to be risk factors [14]. Lin et al. stated that the cephalic vein or jugular vein cut-down technique should be used to avoid the pinch-off syndrome; they emphasized that a port-A catheter should be placed in a natural axis or position, and the junction between the connector and the catheter should not be bent [15]. In this study, we compared the clinical characteristics of patients with or without catheter dislodgement. We found that the only risk factor was the port-A brand (Arrow to Vortex). This is compatible with Wu et al., yet the number of our cases with the Arrow brand was too small to make this conclusion. Interestingly, all episodes of dislodgement in our patients happened after the chemotherapy regimen was completed. The definite cause is not known. During the course of chemotherapy the intravenous dripping has a lower pressure than the bolus flush used after completing the course. This higher pressure is one possible cause. However, there are still many factors to be considered in our study, for example, the installation of the port chamber and the handing protocols of catheters.

Without doubt, early explantation of a port-A catheter will definitely prevent related complications. There are still reasons to retain the device during the post-treatment surveillance since cancer patients require further follow-up to rule out recurrence. In our series, there were 144/330 cases of intentional removal of a port-A catheter. Three of these cases needed re-implantation of the port-A catheter. These three patients were all diagnosed with acute lymphoblastic leukemia relapse. They were off chemotherapy for 19, 27 and 24 months, respectively. The intervals from explantation of the old port-A catheter to implantation of the new one were 59, 66 and 61 months, respectively. Furthermore, it may be a challenge to find new vascular access in patients with previous central venous lines or who had a port-A catheter removed. However, with the improved cure rate of pediatric cancer, we should re-examine how long port-A catheters need to be retained after the completion of chemotherapy.

## Conclusions

The dislodgment rate of port-A catheters in our series was 2.4%. Chest X-rays can rapidly detect the problem. Most catheters were broken at the site of anastomosis. Earlier explantation of a port-A catheter after completion of chemotherapy may avoid dislodgement and migration of the catheter but the possibility of underlying disease recurrence needs to be considered.

## Competing interests

The authors declare that they have no competing interests.

## Authors’ contributions

SCW and CHT collected the data and prepared the manuscript; CPH collected data; SYL, SFK, CCH, YCC and JHC managed the patients; JMS supervised and reviewed the manuscript. All authors read and approved the final version of the manuscript.
